# Exploring the psychometric properties of the premonitory urge for tics scale (PUTS) and its association with psychiatric symptoms in Chinese children with tic disorders

**DOI:** 10.1186/s12887-024-04801-3

**Published:** 2024-05-16

**Authors:** Qiang Ding, Douglas W. Woods, Wen Xu, Ying Zhao, Shuqin Shen, Jinhua Sun

**Affiliations:** 1 Department of Psychological Medicine, Children’s Hospital of Fudan University, National Children’s Medical Center, Shanghai, 201102 China; 2 Department of Psychology, Marquette University, Milwaukee, WI 53233 USA

**Keywords:** Premonitory urge, Tic disorders, PUTS-C, Psychometric properties

## Abstract

**Background:**

The Premonitory Urge for Tics Scale (PUTS) is a common self-report measure of premonitory urges for patients with tic disorders. This study aims to evaluate the Chinese version of the PUTS (PUTS-C) and to explore its association with psychiatric symptoms in Chinese children diagnosed with tic disorders.

**Methods:**

The psychometric evaluation involved 204 outpatients with tic disorders, aged 7–16 years, who were divided into two age groups: (7–10 years, *n* = 103; 11–16 years, *n* = 95).

**Results:**

The PUTS-C demonstrated good internal consistency (McDonald’sω = 0.84) and two-week test-retest reliability (0.76). We observed a statistically significant correlation between the total PUTS-C score and various Yale Global Tic Severity Scale (YGTSS) subscales and total tic severity scores. The PUTS-C score also showed significant correlations with the Children Yale-Brown Obsessive Compulsive Scale (CY-BOCS), Screening Child Anxiety-Related Emotional Disorders (SCARED), and Children’s Depression Inventory (CDI). Notably, premonitory urges independently predicted tic severity, beyond the influence of comorbid symptoms. A two-factor structure of the PUTS-C was identified in the total sample through factor analysis.

**Conclusions:**

The PUTS-C possesses acceptable validity and good reliability. It appears that premonitory urges in Chinese patients with tic disorders are associated with obsessive-compulsive symptoms, anxiety, and depression, but can independently predict tic severity. Specific PUTS-C factors possibly related to motor and vocal tics. Future research should continue to investigate age-related differences and the association with tics and other sensory symptoms.

**Supplementary Information:**

The online version contains supplementary material available at 10.1186/s12887-024-04801-3.

## Introduction

Tic disorders are neurodevelopmental disorders characterized by sudden, rapid, recurrent, nonrhythmic movements or/and vocalizations. Tics can be brief and temporary (such as in Provisional Tic Disorder, PTD), but can also persist for longer than a year, leading to diagnoses of Tourette syndrome (TS) or Persistent Motor or Vocal Tic Disorder (PTD) [1]. Tic disorders are relatively common, with a prevalence rate of 2.5% reported in the mainland of China [2]. Tic disorders are often accompanied by other conditions, particularly obsessive-compulsive disorder (OCD), attention-deficit/hyperactivity disorder (ADHD), anxiety or depression [3].

Premonitory urges (PUs) are distressing physical sensations that immediately precede tics and are experienced by as many as 93% of individuals with TS [4]. Two primary types of premonitory urges are reported by individuals with TS: sensory feelings such as pressure or itching in specific body parts, and mental perceptions such as a sense of incompleteness or things not being quite “right” [5]. Individuals with TS frequently describe premonitory urges as being more upsetting and disruptive than the tics themselves [6]. Thus, these urges are a crucial target of behavior therapy, as they may assist in suppressing the forthcoming tic [5].

The Premonitory Urge for Tics Scale (PUTS) is the most commonly utilized self-report measure to evaluate the intensity of premonitory urges [7]. Studies in Western cultures investigating the psychometric properties of the PUTS have indicated good reliability and validity [8–10]. For instance, Brandt et al. [8] and Raines et al. [9] reported acceptable internal consistency (α = 0.79 andα = 0.82, respectively) and significant correlations with tic severity measures. Openneer et al. [10] confirmed its reliability in a large sample of children and adolescents, highlighting its applicability across different age groups. In the original study [7], the relationship between premonitory urges and tics was investigated by examining the association between the PUTS score and individual items of the Yale Global Tic Severity Scale (YGTSS). Furthermore, the association between premonitory urges and other psychiatric symptoms was explored by correlating the PUTS with specific items of the Child Behavior Checklist (CBCL) and Children’s Yale-Brown Obsessive-Compulsive Scale (CY-BOCS). In the original study, the PUTS demonstrated good stability, high internal consistency, and concurrent and discriminant validity when compared with the YGTSS in the age group of 10 and over. However, these properties could not be confirmed in the younger group (< 10 years). In another study with youth aged 9 to 17, Rozenman et al. [11] found an association between the PUTS score and the intensity of anxiety and somatic/panic symptoms measured by the Screen for Child Anxiety Related Emotional Disorders-Child Version questionnaire (SCARED-C). Performing a thorough analysis of this association for each affected child individually has the potential to provide valuable insights and aid in effectively managing the intensity of premonitory urges during behavioral therapy that incorporates classical conditioning techniques. with the PUTS total score.

To date, limited research has explored the psychometric characteristics of the PUTS scale in a Chinese population, with only a preliminary study probing its psychometric properties [12]. Notably absent in this inquiry is an exploration of how premonitory urges relate to a wider array of psychiatric symptoms beyond tics, such as anxiety, depression, and obsessive-compulsive behaviors. This gap in the literature limits our understanding of the PUTS scale’s full applicability in culturally diverse settings and impedes comprehensive management strategies for tic disorders that consider the interplay between tics, premonitory urges, and comorbid psychiatric conditions. Addressing this void by investigating the associations between premonitory urges and a broader spectrum of symptoms is crucial for advancing our grasp of tic disorders within the Chinese context.

Accordingly, the current study was designed to systematically investigate the psychometric properties of the PUTS-C in a Chinese population. We examine the prevalence and intensity of premonitory urges and assess the internal consistency, test-retest reliability, and concurrent validity of the PUTS-C. Concurrent validity is evaluated by correlating the PUTS-C with the YGTSS motor, vocal, and total tic subscale scores. Associations between the PUTS-C and measures of other symptom domains are also examined. An exploratory factor analysis (EFA) is conducted to determine the latent factor structure of the PUTS-C, and correlations between the resulting factors and measures of psychological functioning are assessed.

## Methods

### Participants

This study involved 210 children and adolescents diagnosed with tic disorders (including provisional tic disorder, chronic motor/phonic tic disorder and Tourette disorder) according to DSM-5 criteria.

The general guideline for sample size determination in scale analysis suggests that the number of participants should be at least 10 times the number of items being analyzed [13]. Given that the PUTS-C comprises 9 items, this implies a minimum required sample size of 90. However, to ensure the robustness of the factor analysis and to accommodate potential attrition and incomplete data, we targeted a larger sample size of 210 children. All participants were recruited from the Department of Psychological Medicine at Children’s Hospital of Fudan University in China between January 2021 and December 2022. To account for potential age-related differences in premonitory urges, the sample was divided into two groups based on age: the “Older group” (*n* = 95) consisted of participants ≥ 11 years old, and the “Younger group” (*n* = 109) consisted of participants ≤ 10 years old, following the definition used by Woods et al. [7] and Raines et al. [9].

The ethics committees of Children’s Hospital of Fudan University approved this study (NO:2021 − 386), and written informed consent was obtained from all the participants and their legal guardians involved in the study.

To ensure consistency in the psychological assessments, a single psychotherapist conducted all clinician-rated assessments in the Department of Psychological Medicine at the Children’s Hospital of Fudan University. The participants included only outpatients who were diagnosed prior to the assessments.

### Measures

#### The premonitory urge for tics Scale (PUTS)

The original PUTS is a self-report rating scale consisting of 9 items, with each rated on a 4-point Likert scale from 1 to 4. Higher scores indicate a greater presence and frequency of premonitory urges. The total score is obtained by summing all items and ranges from 9 to 36.

The Chinese version of the Premonitory Urge for Tics Scale (PUTS-C) was developed through a rigorous process. Initially, the translation of the PUTS-C was performed by a psychiatrist and a psychotherapist at Children’s Hospital of Fudan University. Subsequently, the Chinese translation was back-translated into English by two psychotherapists who were proficient in English. This back-translation was then reviewed by Douglas W. Woods, the original author of the PUTS, and compared with the original English version. Corrections and finalizations to the Chinese version were made based on the feedback provided by the original author.

#### The Yale global tic severity scale (YGTSS)

The YGTSS is used by clinicians to assess the severity of tics experienced by individuals in the past week. This scale is reliable and valid. Clinicians rate tics using a series of 0 to 5-point scales across dimensions of number, frequency, intensity, complexity, and interference. Motor and phonic tics are scored separately. The YGTSS provides three tic severity scores: Total Motor (0–25), Total Phonic (0–25), and Total Tic Score (0–50), which is the sum of the motor and phonic scores. The YGTSS also offers an overall impairment rating, ranging from 0 (indicating no impairment) to 50 (indicating severe impairment), but this was not used in the current study [14].

#### Children Yale-Brown obsessive-compulsive scale (CY-BOCS)

The CY-BOCS is a clinician-rated, semi-structured tool adapted from the Yale-Brown Obsessive-Compulsive Scale (Y-BOCS) for assessing the severity of obsessions and compulsions in children. Each of the 10 items is scored on a 5-point ordinal scale, ranging from 0 (none) to 4 (extreme), with the total score ranging from 0 to 40. The CY-BOCS has demonstrated reliability and validity in children with OCD across various countries. In particular, a study conducted in China with 367 pediatric patients diagnosed with tic disorders, showed that the CY-BOCS had good psychometric properties, with Cronbach’s alpha and test-retest reliability values of 0.81 and 0.82, respectively. The scale also exhibited an acceptable two-factor structure (obsession and compulsion) in patients with tic disorders, further supporting its use in this population [15].

#### Screening child anxiety-related emotional disorders (SCARED)

The SCARED is a self-report measure of anxiety symptoms in children and adolescents, with a total of 41 items. Participants rate the frequency of each symptom on a 3-point scale ranging from 0 to 2, and the relevant item scores are averaged to obtain specific anxiety symptom scores and total anxiety scores. The scale has been used for screening children’s anxiety and has demonstrated good reliability and validity in the context of Chinese culture, according to previous studies [16].

#### The children’s depression inventory (CDI)

The CDI is a self-report questionnaire commonly used to assess depression in children and adolescents, aged 6–17 years. It consists of 27 items, with each item rated on a 3-point scale; 0 indicating no presence of the symptom; 1 indicating mild presence, and 2 indicating the highest severity possible. Total scores range from 0 to 54. Higher scores indicate more severe depressive symptomatology [17].

#### The Swanson, Nolan, and Pelham rating scale (SNAP-IV)

The SNAP-IV is a 26-item scale designed to evaluate symptoms of inattention, hyperactivity/impulsivity, and oppositional behavior in children aged 6 to 17 years. Parents rated the severity of each symptom during the preceding two weeks using a 4-point scale ranging from “not at all” to “very much.” Subscale scores were calculated by summing the item scores, with higher scores indicating more severe symptoms [18].

### Data analysis

Statistical analyses were performed using SPSS software (Version 24.0, SPSS Inc., Chicago, IL, USA).

Before evaluating the psychometric properties of the PUTS-C across different age groups, we conducted analyses to assess potential differences between these groups regarding relevant indicators of psychiatric symptomatology. To compare the younger and older groups in terms of total scores and subscale scores on measures such as YGTSS, CY-BOCS, CDI, SCARED, and SNAP-IV, we initially considered the t-test. However, given the potential concern regarding the normality of the distribution in our sample, we conducted Shapiro-Wilk tests to assess the normality of the data. In cases where the data did not meet the assumptions of normality, non-parametric Mann-Whitney U tests were employed instead of t-tests to ensure the robustness of our findings.

To assess the reliability and validity of the PUTS-C in both the full sample and the younger vs. older samples, several statistical analysis were conducted. Internal consistency was calculated using McDonald’s omega. Test-retest reliability analysis was conducted on 31 participants who completed the two-week retest. Spearman’s rho correlations were used to calculate test-retest reliability.

To test concurrent validity, Spearman’s rho correlations were calculated between PUTS-C total scores, YGTSS motor, vocal, and total tic severity scores, and the different dimensions of tics assessed by the YGTSS (i.e., number, frequency, intensity, complexity and interference).

To examine the correlations of the PUTS-C with symptom severity of OCD, anxiety, depression and ADHD, we calculated Spearman’s rho correlations between PUTS-C total scores and scores on CY-BOCS, SCARED, CDI and SNAP-IV subscales. Subsequently, should significant correlations be found, we intended to conduct stepwise multiple regression analyses. Before proceeding with the stepwise multiple regression analysis, we conducted a thorough examination of the residuals to ensure they conformed to a normal distribution, as well as a collinearity diagnosis to ascertain the independence of our predictors. These analyses would be used to explore whether the PUTS-C scores could predict YGTSS tic severity scores above and beyond the influence of the scores from the CY-BOCS, SCARED and CDI. The purpose of this regression analysis would be to investigate the predictive validity of the PUTS-C with regard to tic severity, independent of comorbid symptoms.

Finally, a principal factor analysis were used to conduct an exploratory factor analysis (EFA) on the PUTS-C items. We also analyzed the Spearman’s rho correlations between the identified factors of the PUTS-C and the YGTSS and CY-BOCS subscale scores, SCARED, CDI, and SNAP-IV subscales.

To explore whether the psychometric properties of the PUTS-C differed across various age groups, we replicated the aforementioned analysis for younger and older subgroups. The aim was to identify any developmental differences in premonitory urges and gain insight into the developmental course of this phenomenon.

## Results

### Characteristics of participants

After excluding six participants who did not complete the survey, the final sample comprised 204 patients. The average age of the participants was 10.65 years (SD = 1.96), with an age range of 7 to 16 years. The majority of the sample were males, accounting for 163 out of the 204 participants.

### Differences between younger and older groups on psychiatric symptoms

Table 1 reports mean scores and analyzes differences between younger and older groups using a variety of measures that assess tic severity and the severity of other psychiatric symptoms. Given the non-normal distribution of our data as indicated by the Shapiro-Wilk test (*p* < 0.05), non-parametric Mann-Whitney U tests were utilized. Significant differences were found in the YGTSS severity scores (U = 6278.5, *p* < 0.01), CY-BOCS (U = 6090, *p* < 0.05), and SCARED (U = 6165.5, *p* < 0.05). No differences emerged between age groups on the CDI total score, SNAP-IV subscales.

**Table 1:** Summary of clinical assessment (Mean ± SD)

Items	Total sample *n* = 204	Older group *n* = 95	Younger group *n* = 109	Mann-Whitney U	*p*
Male/female	163/41	79/16	84/25	/	/
Age	10.65 ± 1.96	12.38 ± 1.34	9.15 ± 0.90	/	/
Total PUTS-C	16.32 ± 5.96	17.77 ± 6.19	15.1 ± 0.49	6524.00	0.001**
YGTSS severity score	21.16 ± 8.71	22.2 ± 9.56	20.27 ± 7.83	6278.5	0.009**
CY-BOCS	17.54 ± 6.76	16.37 ± 10.5	18.79 ± 7.27	6090	0.029*
CDI	53.58 ± 22.60	57.2 ± 24.1	50.42 ± 20.82	5981.5	0.056
SCARED	21.06 ± 13.89	23.66 ± 14.9	18.8 ± 12.56	6165.5	0.019*
SNAP-IV inattention	11.34 ± 6.47	11.97 ± 6.56	10.8 ± 6.38	5675.5	0.236
SNAP-IV hyperactivity/impulsivity	7 ± 5.58	7.03 ± 5.96	6.97 ± 5.25	5005	0.681
SNAP-IV oppositional behaviour	8.17 ± 5.43	8.44 ± 5.28	7.93 ± 5.56	5560.00	0.362

*p < 0.05, **p < 0.01,***p < 0.001;

PUTS-C: Chinese version of Premonitory Urge for Tics Scale; YGTSS: Yale Global Tic Severity Scale; CY-BOCS: Children Yale-Brown Obsessive Compulsive Scale; SCARED: Screening Child Anxiety-Related Emotional Disorders; CDI: Children’s Depression Inventory; SNAP-IV: Swanson, Nolan, and Pelham Rating Scale

### Mean scores of PUTS-C for entire sample and age-related groups

The mean PUTS-C score at initial administration was 16.32 ± 5.96 points (range from 9 to 35). A significant difference was observed between the two age groups (U = 6524, *p* < 0.05); with the older group having a higher urge rating than the younger group. No difference was found (U = 3385.5, *p* = 0.90) between mean PUTS-C scores in males (16.22 ± 5.77) vs. females (16.75 ± 6.77).

To determine if these age-related differences in urge severity were likely due to group differences on the various psychiatric symptom measures, we conducted a Quade Nonparametric ANCOVA comparing PUTS-C scores between older and younger groups, while controlling for YGTSS tic severity, CY-BOCS, CDI, and SCARED total scores. After controlling for these differences, there was no significant difference between two age groups (F_(1,202)_ = 3.13, *p* = 0.08).

### Psychometric properties of PUTS-C

The PUTS-C showcased commendable psychometric strength, evidenced by an overall McDonald’s omega of 0.84, indicating high internal consistency across age groups, with older (ω = 0.82) and younger (ω = 0.84) participants showing similar reliability. The scale’s temporal stability was confirmed through a two-week test-retest reliability of 0.76 (*n* = 31). Construct validity was supported by its correlations with the YGTSS (see Table 2): total tic severity (ρ = 0.22, *p* < 0.01), motor scores (ρ = 0.18, *p* < 0.01), vocal scores (ρ = 0.17, *p* < 0.05), complexity (ρ = 0.22, *p* < 0.01), and interference (ρ = 0.21, *p* < 0.01), though not with tic frequency. Distinct patterns emerged between age groups; older children’s PUTS-C scores correlated with YGTSS number subscale (ρ = 0.20, *p* < 0.05) and motor scores (ρ = 0.22, *p* < 0.05), whereas younger children’s scores significantly associated with YGTSS severity (ρ = 0.20, *p* < 0.05), complexity (ρ = 0.23, *p* < 0.05), and interference (ρ = 0.20, *p* < 0.05).

**Table 2:** Correlations between the PUTS-C total score and the YGTSS subscales for the total sample and different age groups

Subjects	YGTSS severity score	YGTSS motor score	YGTSS vocal score	YGTSS Subscales				
				Number	Frequency	Intensity	Complexity	Interference
Total sample (*N* = 204)	0.22**	0.18**	0.17*	0.21**	0.11	0.16*	0.22**	0.21**
Older group (*N* = 95)	0.17	0.22*	0.12	0.20*	0.16	0.12	0.16	0.14
Younger group (*N* = 109)	0.20*	0.08	0.18	0.19	0.03	0.15	0.23*	0.20*

*p < 0.05, **p < 0.01,***p < 0.001;

PUTS-C: Chinese version of Premonitory Urge for Tics Scale; YGTSS: Yale Global Tic Severity Scale

### Associations of the PUTS-C with other symptom domains

For the entire sample of 204 youths, there were significantly positive correlations between the total PUTS-C score and the CY-BOCS (ρ = 0.40, *p* < 0.001), CDI (ρ = 0.46, *p* < 0.001), and SCARED (ρ = 0.48, *p* < 0.001). For subscales of the SNAP-IV, there were no significant correlations with PUTS-C (*p >* 0.05).

Given the significantly positive correlations between PUTS-C and the CY-BOCS, SCARED, and CDI, it is possible that any relationships between the PUTS-C and YGTSS might be the result of mutual relationship with one of these other measures and thus call into question the use of the PUTS-C as a valid measure for rating a phenomenon specific to tics. As such, we conducted stepwise multiple regression to examine the relationship between total PUTS-C scores and YGTSS tic severity scores, after controlling for CY-BOCS, SCARED and CDI scores. The Normal P-P Plot of Regression Standardized Residual indicated that the residuals from our model approximated a normal distribution, with the data points closely hugging the diagonal line and only minor, non-significant deviations observed (see Supplementary Fig. 1). Additionally, the collinearity diagnostics, including the Variance Inflation Factor (VIF), revealed that all predictors had VIF values well below the commonly used threshold of 5, indicating a lack of problematic multicollinearity. The PUTS-C (β = 0.22, *p =* 0.002) predicted tic severity (R^2^ = 0.047, F_(1,202)_ = 9.98, *p* = 0.002), even after controlling for CY-BOCS (β = 0.03, *p* = 0.704), SCARED (β = 0.09, *p* = 0.275) and CDI (β = 0.08, *p* = 0.297), suggesting the PUTS-C is a unique predictor of tic severity. Combined, these results indicate the PUTS-C demonstrates acceptable validity.

Consistent with prior subgroup differences, separate patterns of correlations emerged between the total PUTS-C score and the CY-BOCS, CDI, SCARED, and SNAP-IV scores when examining the younger and older groups. For both the younger and older groups, the PUTS-C was positively correlated with the CY-BOCS (ρ_older_ = 0.41, *p* < 0.01; ρ_younger_ = 0.36, *p* < 0.01), CDI (ρ_older_ = 0.45, *p* < 0.01; ρ_younger_ = 0.41, *p* < 0.001) and SCARED (ρ_older_ = 0.46, *p* < 0.01; ρ_younger_ = 0.43, *p* < 0.001). For subscales of the SNAP-IV, there were no significant correlations with PUTS-C for either the younger group or the older group (see Table 3). These findings suggest that urges are related to, but not the same phenomenon as internalizing symptomatology.

**Table 3:** Correlations between the PUTS-C total score and CY-BOCS, CDI, SCARED, SNAP-IV for the total sample and different age groups

Validity	Total sample *n* = 204	Older group *n* = 95	Younger group *n* = 109
CY-BOCS	0.40***	0.36***	0.41***
CDI	0.46***	0.41***	0.45***
SCARED	0.48***	0.43***	0.46***
SNAP-IV inattention	0.13	0.06	0.17
SNAP-IV hyperactivity/impulsivity	0.06	0.04	0.08
SNAP-IV oppositional behaviour	0.09	0.04	0.1

*p < 0.05, **p < 0.01,***p < 0.001

PUTS-C: Chinese version of Premonitory Urge for Tics Scale; CY-BOCS: Children Yale-Brown Obsessive Compulsive Scale; SCARED: Screening Child Anxiety-Related Emotional Disorders; CDI: Children’s Depression Inventory; SNAP-IV: Swanson, Nolan, and Pelham Rating Scale

### Factor structure of PUTS-C

Following recommendations for initial inter-item correlations (see Supplementary Table 1) [19] and taking into account the potential impact of sample size [20], the exploratory factor analysis (EFA) of the PUTS-C was conducted without differentiating between age groups. This analysis, employing maximum likelihood with direct oblimin rotation on the 9-item scale, demonstrated suitability (KMO = 0.87, Bartlett’s test χ^2^_(36)_ = 576.25, *p* < 0.001), and delineated two distinct factors explaining 55.6% of the variance: Factor 1 (Items 5, 6, 7, and 8) and Factor 2 (Items 1, 2, 3, 4, and 9, see Supplementary Table 2). Subsequent correlation analyses illustrated Factor 1’s significant association with YGTSS motor scores (ρ = 0.23, *p* < 0.01), and Factor 2’s with vocal scores (ρ = 0.2, *p* < 0.01), alongside both factors correlating with YGTSS severity and various tic dimensions (number, intensity, complexity, interference), and with CY-BOCS, SCARED, and CDI scores (see Table 4).

**Table 4:** Correlations between the PUTS-C two-factor dimensions score and the YGTSS subscale for the total sample

subjects		YGTSS severity score	YGTSS motor score	YGTSS vocal score	YGTSS subscale				
					Number	Frequency	Intensity	Complexity	Interference
Total sample (*N* = 204)	factor1	0.23***	0.23**	0.10	0.20**	0.1	0.15*	0.20**	0.19**
	factor2	0.17*	0.11	0.20**	0.18*	0.1	0.15*	0.21**	0.21**
		CY-BOCS	SCARED	CDI	SNAP-IV				
					Inattention	Hyperactivity /impulsivity		Oppositional behaviour	
	factor1	0.34***	0.43***	0.40***	0.11	0.06		0.14*	
	factor2	0.38***	0.46***	0.43***	0.13	0.06		0.05	

*p < 0.05, **p < 0.01,***p < 0.001

PUTS-C: Chinese version of Premonitory Urge for Tics Scale; YGTSS: Yale Global Tic Severity Scale; CY-BOCS: Children Yale-Brown Obsessive Compulsive Scale; SCARED: Screening Child Anxiety-Related Emotional Disorders; CDI: Children’s Depression Inventory; SNAP-IV: Swanson, Nolan, and Pelham Rating Scale

## Discussion

This study systematically investigated the psychometric properties of the PUTS in Chinese children with tic disorders, unveiling the dynamics of premonitory urges within this group. Our initial analysis revealed age-related differences in premonitory urge severity, contrasting with earlier findings that detected no such variations in PUTS scores [12]. However, these differences dissipated after adjusting for psychiatric symptoms, suggesting that what appeared as age-related variations in Premonitory Urge severity may actually reflect the influence of psychiatric symptoms. This insight, stemming from our use of 11 years as the age cutoff compared to the previous standard of 10 years [12], underscores the necessity of considering developmental stages and psychiatric contexts in premonitory urges research.

### Psychometric properties of PUTS-C

The PUTS-C demonstrated robust internal consistency across age groups in our study, diverging from Woods et al.‘s findings of age-related differences in Cronbach’s alpha values [7]. This discrepancy may arise from differences in study settings; our clinical context might have facilitated more consistent responses across age groups, potentially with parental assistance for younger children, aligning with findings by Openneer et al. [10] and Li et al. [12]. Further exploration is warranted to clarify these differences.

Concurrently, PUTS-C scores correlated weakly but significantly with YGTSS scores, supporting the notion that they measure related yet distinct constructs, echoing broader research indicating a biological divergence between premonitory urges and tic expressions [5, 22–25]. Notably, our study identified a significant correlation with tic intensity, adding to evidence that stronger premonitory urges may predict more severe tic manifestations [26, 27].

Age-related differences in PUTS-C and YGTSS correlations were observed, with stronger associations in younger children. This may reflect cultural influences on how bodily sensations are reported, with Chinese youths potentially underreporting premonitory urges due to cultural norms of emotional restraint [28]. Alternatively, younger children’s difficulty in distinguishing between urges and tics [29] could amplify perceived correlations, underscoring the importance of tailored age-appropriate guidance in using the PUTS-C.

### Associations of the PUTS-C with other symptom domains

The significant correlations we observed between PUTS-C scores and measures of OCD (CY-BOCS), depression (CDI), and anxiety (SCARED) not only underscore the complex interplay between premonitory urges and a spectrum of psychiatric symptoms but also hint at the underlying psychophysiological mechanisms that could be common across these disorders [7, 29, 30]. This convergence suggests a shared mechanism involving heightened sensitivity to bodily sensations or an enhanced interoceptive awareness. Such awareness could predispose individuals to more acutely perceive premonitory urges and experience higher levels of anxiety, depression, and OCD symptoms, indicating a psychophysiological substrate that spans across different symptom domains [31].

Further exploration through the lenses of psychological stress and cognitive-behavioral models offers deeper insights into these correlations. According to the psychological stress model, the persistent nature of premonitory urges and the efforts to suppress them generate significant stress, which in turn exacerbates symptoms of anxiety and depression. This underscores a crucial psychophysiological linkage among these symptom domains [32]. Concurrently, the cognitive-behavioral model elucidates the processes through which individuals perceive, interpret, and manage premonitory urges. It posits that individuals’ interpretations of and responses to these sensations—such as negative self-evaluations and concerns about the social repercussions of tic behaviors—can intensify psychiatric symptoms. This underscores the potential of cognitive-behavioral interventions to modify maladaptive beliefs and enhance coping strategies, thereby alleviating psychophysiological symptoms and improving mental health outcomes [33].

Moreover, when considering the developmental aspects of these experiences, our analysis reveals that the interconnection between premonitory urges, anxiety, and depression is more pronounced in younger children. This suggests that developmental factors significantly influence how these sensations are perceived and reported, with younger children potentially experiencing a less differentiated sensation of bodily states or a limited capacity to verbally articulate these experiences. This observation aligns with the cognitive-behavioral model’s emphasis on cognitive and emotional regulation development, highlighting a need for age-specific investigations into interoceptive discrimination and the evolution of coping strategies.

Additionally, the specificity of certain PUTS-C items—like those describing feelings of being “wound up” or experiencing “excessive energy”—may inadvertently overlap with symptoms commonly associated with anxiety or ADHD. This raises important questions regarding the PUTS-C’s specificity in distinguishing sensory experiences inherent to tic disorders from those indicative of comorbid conditions [11]. It points to the necessity for further research into the relationship between premonitory urges and sensory symptoms, aiming to refine diagnostic tools and therapeutic approaches through a more integrated understanding of the psychological and physiological dimensions of tic disorders.

### Factor structure of the PUTS-C

Exploratory factor analysis of the PUTS-C in our study identified a two-factor structure, aligning with findings from Raines et al. [9] and Brandt et al. [8]. The first factor comprises items assessing sensations like itchiness, pressure, tension, and a sense of incompleteness, which subside post-tic. This factor closely mirrors the qualitative aspect of premonitory sensations reported in previous studies.

The second factor, involving sensations of incompleteness and a need for energy release, correlates with the frequency and universality of these urges before tics. Notably, our analysis suggests a novel differentiation: the first factor primarily associates with motor tics, while the second links more with vocal tics. This distinction underscores the nuanced relationship between the nature of premonitory urges and tic types, indicating motor tics might be preceded by urges of pressure or energy, and vocal tics by sensations of itchiness or tightness.

### Limitations and future directions

The current study has certain limitations that should be acknowledged. First, we did not conduct a formal structured assessment of comorbid conditions during participant recruitment, which calls for caution in generalizing the results. It would be beneficial for future research to conduct a comparative analysis of the response to the PUTS-C among children and adolescents with diverse comorbidity profiles. Additionally, our sample was limited to children and adolescents, and expanding the sample to include Chinese adults diagnosed with tic disorders would be valuable for future studies. Despite these limitations, our study demonstrates strong psychometric properties, particularly in terms of reliability and construct validity, of the PUTS-C. The findings suggest that the PUTS-C is a reliable tool for assessing premonitory urges in individuals with tic disorders in Chinese clinical and research settings.

## Conclusion

The study demonstrates that the PUTS-C exhibits good reliability and acceptable validity in Chinese children with tic disorders. Furthermore, the research establishes a significant relationship between premonitory urges and symptoms of obsessive-compulsive disorder, anxiety, and depression within this population. To gain deeper insights, future investigations should explore the application of the PUTS-C across various age groups, aiming to elucidate age-related variations in premonitory urges and their association with tics and other sensory symptoms.

### Electronic supplementary material

Below is the link to the electronic supplementary material.

Supplementary Material 1

## Data Availability

The datasets used and/or analysed during the current study are available from the corresponding author on reasonable request.
