# Supplementary material for: Exploring the psychometric properties of the premonitory urge for tics scale (PUTS) and its association with psychiatric symptoms in Chinese children with tic disorders
Source: BMC Pediatr. 2024 May 16;24:341. doi: 10.1186/s12887-024-04801-3 (PMC11097492; doi:10.1186/s12887-024-04801-3)
Supplement: Supplementary file 1 — Supplementary Material 1 [file 12887_2024_4801_MOESM1_ESM.docx]

**Supplementary Materials**

**Table 1** PUTS-C Inter-item Correlations-Total sample(n=204)

| PUTS | PUTS-1 | PUTS-2 | PUTS-3 | PUTS-4 | PUTS-5 | PUTS-6 | PUTS-7 | PUTS-8 | PUTS-9 |
| --- | --- | --- | --- | --- | --- | --- | --- | --- | --- |
| PUTS-1 | 1 |  |  |  |  |  |  |  |  |
| PUTS-2 | 0.20** | 1 |  |  |  |  |  |  |  |
| PUTS-3 | 0.36*** | 0.41*** | 1 |  |  |  |  |  |  |
| PUTS-4 | 0.22** | 0.31*** | 0.31*** | 1 |  |  |  |  |  |
| PUTS-5 | 0.17** | 0.24*** | 0.29*** | 0.36*** | 1 |  |  |  |  |
| PUTS-6 | 0.15* | 0.41*** | 0.31*** | 0.33*** | 0.40*** | 1 |  |  |  |
| PUTS-7 | 0.31*** | 0.40*** | 0.38*** | 0.34*** | 0.45*** | 0.56*** | 1 |  |  |
| PUTS-8 | 0.26*** | 0.44*** | 0.39*** | 0.40*** | 0.47*** | 0.58*** | 0.71** | 1 |  |
| PUTS-9 | 0.27*** | 0.35*** | 0.37*** | 0.43*** | 0.38*** | 0.39*** | 0.35** | 0.47** | 1 |

**p*< 0.05, ***p*<0.01,****p*<0.001

PUTS-C: Chinese version of Premonitory Urge for Tics Scale

**Table 2** Maximum likelihood exploratory factor analysis with structure and pattern matrices for PUTS-C

|  | Total sample(n= 204) | | | | |
| --- | --- | --- | --- | --- | --- |
|  | Structure Matrix | | Pattern Matrix | | Communalities |
|  | factor 1 | factor 2 | factor 1 | factor 2 |  |
| 1. Right before I do a tic, I feel like my insides are itchy | 0.32 | **0.53** | -0.08 | **0.58** | 0.28 |
| 2. Right before I do a tic, I feel pressure inside my brain or body | 0.52 | **0.57** | 0.26 | **0.39** | 0.36 |
| 3. Right before I do a tic, I feel “wound up” or tense inside | 0.43 | **0.65** | -0.02 | **0.67** | 0.43 |
| 4. Right before I do a tic, I feel like something is not “just right” | 0.47 | **0.55** | 0.17 | **0.44** | 0.32 |
| 5. Right before I do a tic, I feel like something is not complete | **0.54** | 0.45 | **0.43** | 0.16 | 0.31 |
| 6. Right before I do a tic, I feel like there is energy in my body that needs to get out | **0.70** | 0.50 | **0.67** | 0.05 | 0.49 |
| 7. I have these feelings almost all the time before I do a tic | **0.83** | 0.52 | **0.88** | -0.08 | 0.69 |
| 8. These feelings happen for every tic I have | **0.85** | 0.58 | **0.84** | 0.01 | 0.72 |
| 9. After I do the tic, the itchiness, energy, pressure, tense feelings, or feelings that something is not ‘‘just right’’ or complete go away, at least for a little while | 0.47 | **0.56** | 0.16 | **0.46** | 0.33 |
| Eigen value | 3.97 | 1.03 |  |  |  |
| Common variance (%) | 44.09 | 11.47 |  |  |  |
| KMO | 0.87 | |  | |  |
|  |  |  |  |  |  |

Bold items indicate values which loaded onto a factor.

PUTS-C: Chinese version of Premonitory Urge for Tics Scale


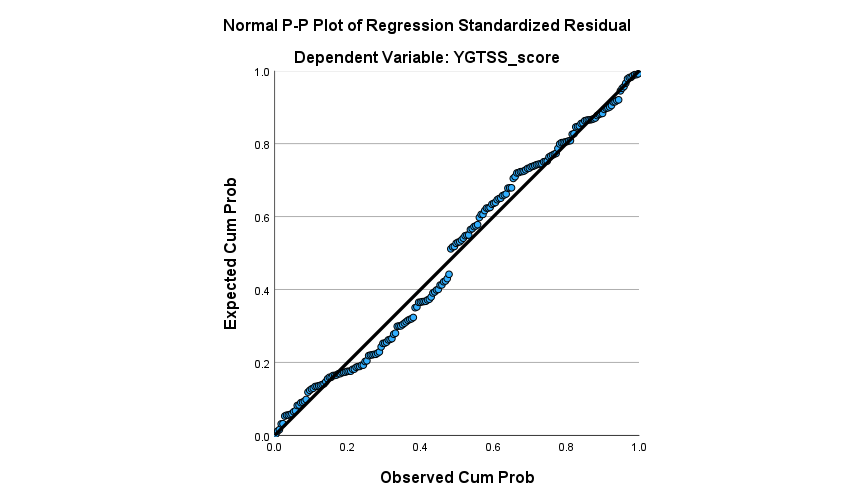


**Figure1** Normal Probability Plot of Standardized Residuals for Tic Severity Score Regression Model
